# Editorial: Alveolar Macrophages in Lung Inflammation and Resolution

**DOI:** 10.3389/fimmu.2019.02275

**Published:** 2019-09-24

**Authors:** Guochang Hu, John W. Christman

**Affiliations:** ^1^Departments of Anesthesiology and Pharmacology, University of Illinois College of Medicine, Chicago, IL, United States; ^2^Pulmonary, Critical Care, and Sleep Medicine, Ohio State University Wexner Medical Center, Davis Heart Lung Research Institute, Columbus, OH, United States

**Keywords:** lung injury, inflammation, alveolar macrophage, immunity, phenotype

Macrophages and macrophage-like cells are present in all mammalian organs with substantial heterogeneity and phenotypic specialization that is regulated in tissue-specific manner. In the lung, there are two distinct macrophage populations: alveolar macrophages, which are in close contact with the type I and II epithelial cells of alveoli (1); and interstitial macrophages, which reside in the parenchyma between the microvascular endothelium and alveolar epithelium (2). Alveolar macrophages derive from yoke sac procurers of fetal monocytes, which populate the alveoli shortly after birth and persist over the lifespan via self-renewing embryo-derived populations independently of bone marrow contribution (3–5). Following inflammatory insults, bone marrow-derived monocytes are recruited to the lung and differentiate into alveolar macrophages (6–8). Terminal differentiation and maturation of lung macrophages is dependent on granulocyte macrophage-colony stimulating factor and transduced by the transcription factors, Pu.1 (9). The functional phenotype of alveolar macrophages is modulated by the unique microenvironment of the lung that includes intimate contact with epithelial cells, high oxygen tension, and exposure to surfactant-rich fluid. Alveolar macrophages are critical for tissue homeostasis, host defense, clearance of surfactant and cell debris, pathogen recognition, initiation and resolution of lung inflammation, and repair of damaged tissue (10). Under physiological conditions, alveolar macrophages produce low levels of inflammatory cytokines, maintain high phagocytic activity, and generally suppress inflammation and adaptive immunity (1).

Alveolar macrophages are the first line of defense against pollutants and pathogenic microbes that initiate an innate immune response in the lung. Two phenotypes of alveolar macrophages have been identified: classically activated macrophage (M1 macrophage) and alternatively activated macrophage (M2 macrophage). M1 macrophages respond to microbial factors and Th1 proinflammatory cytokines to exhibit glycolytic metabolism that is associated with inflammatory cytokine release, enhanced bacterial killing, and the recruitment of immune cells into the lung parenchyma and alveolus. In comparison, M2 macrophages are induced by exposure to the Th2 cytokines to undergo oxidative metabolism that is associated with anti-inflammatory cytokine release, phagocytosis of apoptotic cells (efferocytosis) and collagen deposition that contribute to the resolution of inflammation and repair of damaged tissues (11, 12). The protean role of alveolar macrophage in the pathogenesis and resolution of lung inflammation is dependent on their ontogeny and the microenvironment associated with various noxious stimuli (13). Due to their remarkable plasticity, alveolar macrophages are highly specialized in reacting to environmental signals leading to rapid and reversible changes in their inflammatory phenotype (14). In response to damage-associated molecular patterns, pathogen-associated molecular patterns, cytokines, growth factors, and other mediators released in the microenvironment, alveolar macrophages are phenotypically and functionally modified to adopt pro-inflammatory, pro-fibrotic, anti-inflammatory, anti-fibrotic, pro-asthmatic, pro-resolving, or tissue regenerating properties (15, 16). The transcriptome and epigenetic landscape of alveolar macrophages are determined by the lung microenvironment (17). During lung inflammation, macrophages also constantly communicate with and epithelial cells, microvascular endothelial cells, neutrophils, macrophages, lymphocytes, fibroblasts, and stem or tissue progenitor cells to regulate lung homeostasis and innate and adaptive immunity against pathogens (18–22). The polarization states of alveolar macrophages are not mutually exclusive and cells can exhibit elements of both M1 and M2 macrophages simultaneously depending on environmental signals (23). The high plasticity of macrophages makes it difficult to distinguish the specific subpopulations. Cell surface markers and transcriptional and epigenetic profiles are a focus of current research in order to identify the unique role of the distinct macrophage populations and activation states in lung injury and repair (24, 25).

This collection of published articles is comprised of a series of reviews and original research papers underlining the role of alveolar macrophages in lung inflammation. Through a systematic review and meta-analysis of 22 studies using different animal species including rats, mice, rabbits, dogs, pigs, and sheep, Liu et al. concluded that fibrinolytic therapy significantly improved gas exchange, reduced lung inflammatory injury, and prolonged survival in preclinical animal models. Feller et al. showed that persistent cigarette smoking activated non-canonical Wnt family member 5a signaling which down-regulated peroxisome proliferator-activated receptor gamma expression, leading to polarization of macrophages from anti-inflammatory M2 to pro-inflammatory M1 phenotype, lung inflammation and ultimate chronic obstructive pulmonary disease. Tissue hypoxia is a common microenvironmental feature of sepsis and other inflammatory diseases. Wu et al. demonstrated that the expression of inflammatory genes including tumor necrosis factor α, interleukin-1β, and interleukin-6, Toll-like receptor 4 in the alveolar macrophages was enhanced upon acute hypoxia exposure during endotoxemia in rats. Lee et al. discussed the recent findings on the interaction between alveolar macrophages and lung epithelial cells via extracellular vesicles and extracellular vesicle-containing microRNAs. Bidirectional paracrine cross-talk between macrophages and epithelium via extracellular vesicle-mediated signaling may trigger an inflammatory cascade in the lung. In a mouse model of bleomycin-induced pulmonary fibrosis, Elewa et al. reported that the subpopulations of CD80^+^ M1 macrophages increased and there was a significantly positive correlation in the number of infiltrated macrophages between the lungs and mediastinal fat-associated lymphoid clusters. This study suggests that mediastinal fat-associated lymphoid clusters may play an essential role in the progression of lung inflammatory diseases. Finally, McCubbrey et al. assessed the efficiency and specificity of commonly used mouse strains targeting lung macrophages. The specificity for targeting lung macrophages with lysozyme M-Cre is higher than with colony stimulating factor 1 receptor-Cre. There was highly efficient gene depletion in alveolar macrophages and interstitial macrophages with either lysozyme M-Cre or colony stimulating factor 1 receptor-Cre. The chemokine (C-X3-C motif) receptor 1-estrogen receptor Cre and the reverse tetracycline-controlled transactivator protein under the control of the human CD68 promoter inducible systems mainly targeted interstitial macrophages and trafficking monocytes, but were unable to delete floxed genes in alveolar macrophages.

In summary, we anticipate that the collection of reviews and original articles will serve as inspiration of future research to identify how the specialized microenvironment of the airspaces following injury drives the polarization of alveolar macrophages which regulates lung inflammation and resolution. Advances in the understanding of the function and regulatory mechanisms of alveolar macrophage may provide insights that could lead to novel therapies for pulmonary diseases via specifically targeting the subpopulations of alveolar macrophages.

## Author Contributions

All authors listed have made a substantial, direct and intellectual contribution to the work, and approved it for publication.

### Conflict of Interest

The authors declare that the research was conducted in the absence of any commercial or financial relationships that could be construed as a potential conflict of interest.
